# Scattering-type scanning near-field optical microscopy with reconstruction of vertical interaction

**DOI:** 10.1038/ncomms9973

**Published:** 2015-11-23

**Authors:** Le Wang, Xiaoji G. Xu

**Affiliations:** 1 Department of Chemistry, Lehigh University, 6 E Packer Avenue, Bethlehem, Pennsylvania 18015, USA

## Abstract

Scattering-type scanning near-field optical microscopy provides access to super-resolution spectroscopic imaging of the surfaces of a variety of materials and nanostructures. In addition to chemical identification, it enables observations of nano-optical phenomena, such as mid-infrared plasmons in graphene and phonon polaritons in boron nitride. Despite the high lateral spatial resolution, scattering-type near-field optical microscopy is not able to provide characteristics of near-field responses in the vertical dimension, normal to the sample surface. Here, we present an accurate and fast reconstruction method to obtain vertical characteristics of near-field interactions. For its first application, we investigated the bound electromagnetic field component of surface phonon polaritons on the surface of boron nitride nanotubes and found that it decays within 20 nm with a considerable phase change in the near-field signal. The method is expected to provide characterization of the vertical field distribution of a wide range of nano-optical materials and structures.

## Abstract (web summary)

Conventionally, scattering-type scanning near-field optical microscopy does not provide information on the vertical characteristic of near-field responses. Here, Xu *et al*. develop a method to reconstruct the vertical interaction response between the tip and the sample using this near-field technique.

## Graphical abstract

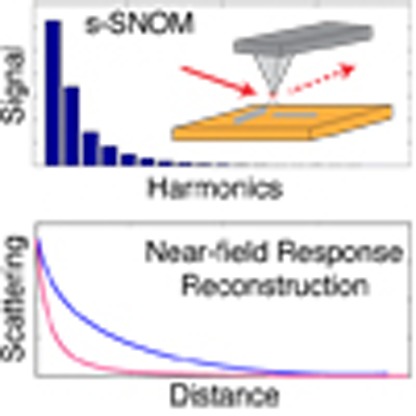

Scattering-type near-field microscopy provides a general way to overcome the optical diffraction limit on the spatial resolution for surface spectroscopic analysis^1^. Unlike various approaches based on fluorescence super resolution^2^,^3^,^4^, scattering type near-field techniques do not require attachment of fluorophores and can operate with wide range of light wavelengths. In particular, infrared scattering-type scanning near-field optical microscopy (s-SNOM, also known as apertureless near-field scanning optical microscopy)^5^,^6^ is proven to be a widely applicable method for surface spectroscopic imaging^7^ at a spatial resolution of <20 nm.

Scattering-type scanning near-field optical microscopy uses a sharp metallic or metal-coated tip to enhance and probe the local electric field in a close proximity to the sample. With an external light source at a frequency of choice, the metallic tip scatters the light according to its polarizability. The presence of the sample under the metallic tip modifies the polarizability of the tip according to the dielectric function of the sample, which is affected by the presence of electronic, vibrational or phonon resonances^8^,^9^,^10^,^11^. By scanning the metallic probe over the sample with an atomic force microscope (AFM) in intermittent contact mode (also known as the tapping mode), one can use the s-SNOM signal obtained through lock-in detection to form an image or further process the signal to obtain spectra of the sample^8^,^12^,^13^,^14^. Scattering-type scanning near-field optical microscopy has been successfully applied to *in situ* spectral imaging of various features in condensed phase materials, including the metal-to-insulator transition of correlated electronic materials^15^, metallic plasmonic structures^16^, surface phonon polaritons of silicon carbide^17^ and boron nitride^18^,^19^,^20^, graphene plasmons^21^,^22^, block copolymers^23^,^24^, peptide and proteins^25^,^26^,^27^ and charge carriers of a topological insulator^28^. In these applications, s-SNOM produces a two-dimensional image of the sample surface based on the tip scattering signal at a specific position. Generally, the use of the vertical near-field behaviour along the direction normal to the plane of the sample surface remains limited in s-SNOM^29^,^30^. On the other hand, plasmonic or polaritonic materials are associated with local electric field bound to the range near surface. An extension of s-SNOM, which is capable of obtaining information on the vertical characteristic of the near-field, shall provide access to studying surface-confined phenomena of such materials.

The challenge of current s-SNOM methods to provide the vertical interaction is indirectly due to its large far-field scattering background, that is, reflection or scattering in the absence of short-range tip–sample interaction from the tip shaft, cantilever, and because of roughness of the sample. Because s-SNOM is an elastic scattering method, the light scattered by the apex of a sharp metallic tip held at a static position above the sample is not distinguishable in frequency from diffused or scattered light from the surroundings. In comparison, the near-field signals obtained in inelastic scattering method such as tip-enhanced Raman spectroscopy^31^ can be separated from the background with a band-pass filter, because the signal is Raman-shifted from the incident light. The distance dependence of tip-enhanced Raman spectroscopy is obtained by steadily retracting the tip from the sample surface while recording the Raman signal. In s-SNOM, to remove the large background, the probe tip is kept sinusoidally oscillating above the sample in the tapping mode operation, and the near-field signal is extracted at the multiple of the tip oscillation frequency with a lock-in amplifier. Non-fundamental harmonic demodulations (*n*≥2) are used^8^. In s-SNOM, an approach curve (often called ‘the *Z*-plot') can be acquired by moving the oscillating AFM tip towards the sample surface and recording the lock-in demodulation signal until the oscillation amplitude reaches the amplitude set point^9^. However, approach curves do not provide a straightforward description of the vertical dependence of the near-field signal. They are obtained from a convoluted signal generation mechanism that depends on the tip oscillation amplitude and the order of lock-in demodulation. Consequently, approach curves lack a direct and unambiguous relationship to the vertical characteristics of confinement of the near-field interaction between the tip and sample.

In this article, we develop a method to reconstruct the vertical characteristics of the interaction between the tip and the sample in s-SNOM. This approach is inspired by a technique used in dynamic force microscopy for reconstruction of a nonlinear property^32^. With the access to the vertical interaction response, we investigate the bound-field component of surface phonon polaritons (SPhPs) in boron nitride nanotubes (BNNTs). The presence of SPhPs leads to the strengthening of the near-field interaction in the 20 nm range from the surface, and is accompanied by a considerable phase shift of near-field scattering signal.

## Results

### Parameter dependence of the shape of approach curves

Supplementary Fig. 1 shows the calculated approach curves for different combinations of tip oscillation amplitudes and orders of lock-in demodulation. These curves display different shapes on the same sample. This behaviour illustrates one of the challenges of the approach curve method to study the vertical characteristics of near-field response on a specific sample.

### Vertical reconstruction of the near-field interaction

**Figure 1:**
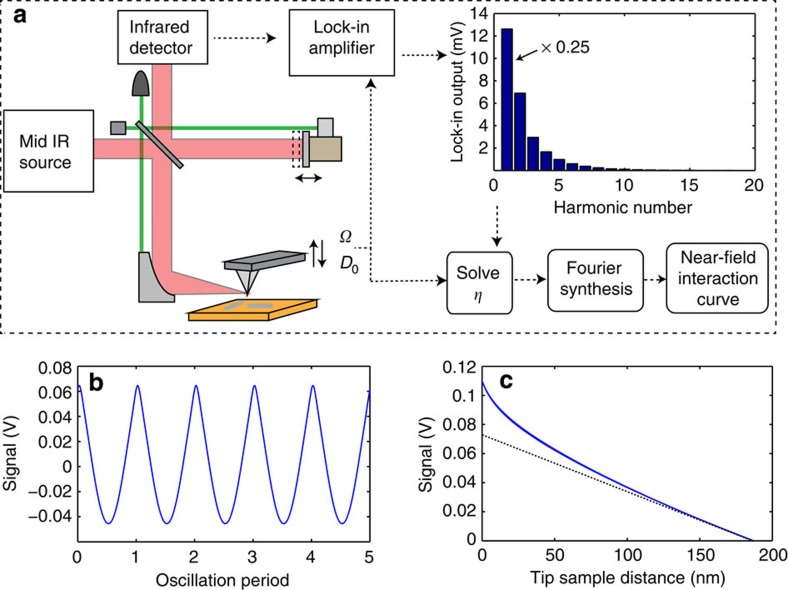
Scheme of method of the near-field interaction reconstruction. (**a**) The scheme of the experimental setup and operating principle. The left side shows the s-SNOM apparatus with homodyne detection. The inset on the right side shows the amplitude of lock-in demodulation of 18 harmonics from experimental measurement of Pt-coated tip and a gold substrate. (**b**) Reconstructed raw s-SNOM waveform. (**c**) Scattering-type scanning near-field optical microscopic interaction curve. Dashed line shows the linear trend of the background, which is a characteristic of the far-field contribution.

The method to reconstruct the vertical characteristic of near-field interaction involves five steps: (i) the collection of multiple harmonics with a lock-in detection in an s-SNOM apparatus; (ii) conversion of tip oscillatory phase to tip–sample distance; (iii) finding a first harmonic contribution coefficient to remove the non-near-field background; (iv) Fourier synthesis of lock-in harmonics; and (v) reconstruction of the relation between tip–sample distance and scattering signal. The elements of the method are shown in Fig. 1a. A custom-built s-SNOM apparatus consists of a mid-infrared light source, a tapping mode AFM, an asymmetric Michelson interferometer and an infrared detector. The signal from the detector carries the near-field response as well as non-near-field background. The near-field response refers to the change of tip-scattering signal due to the modification of the effective tip polarizability through short-range tip–sample interaction. It is present when the tip and sample are in close range, and it relies on the induction and detection of the evanescent field by the metallic tip. The non-near-field background refers to the signal that has long-range characteristics, including the far-field scattering from the cantilever and the rough sample surface. Note that the non-near-field background also contains the non-evanescent component of the near field established by the sample and the tip, which does not carry high spatial frequencies that near-field methods rely on. As the tip is harmonically oscillating at a drive frequency *Ω* above the sample, the combined signal from near-field scattering and non-near-field background exhibits periodical oscillations at the same frequency. The signal is then coupled into a multi-channel lock-in amplifier to collect sufficient numbers of harmonics of the demodulation amplitude *S*_*n*_, together with the lock-in phase *Φ*_*n*_. The subscript *n* refers to the order of lock-in harmonic demodulation, with corresponding reference frequency at *nΩ*. In our implementation, we collected 18 lock-in harmonics. The inset of Fig. 1a shows an example of an experimentally collected spectrum of *S*_*n*_ for a Pt-coated tip on a gold substrate (lock-in phase is shown in Supplementary Fig. 2). The necessary number of lock-in harmonics to be collected is determined by rate of change of the near-field signal. We have found that 18 harmonics are sufficient for the reconstruction (see Supplementary Fig. 3).

The waveform *S*(*t*) of the s-SNOM signal can be obtained straightforwardly through the procedure of Fourier synthesis (equation (1)) using a set of parameters-measured lock-in demodulation amplitude *S*_*n*_ and lock-in phase *Φ*_*n*_


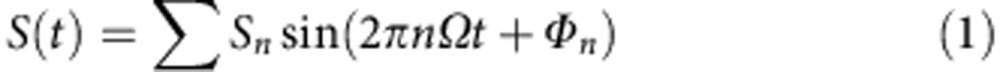


where *t* represents time. The instantaneous tip–sample distance *D*(*t*) is given in equation (2). A standard AFM Z-ramp procedure is used to determine the oscillation amplitude *D*_0_ (see Supplementary Fig. 4).


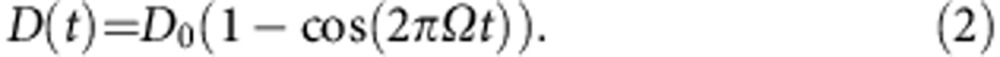


Equation (2) assumes that the apex of the metallic tip touches the surface of the sample at the lower turning point of the oscillation. After solving for *t*, we obtain the relation between *D* and *S* from equation (3)


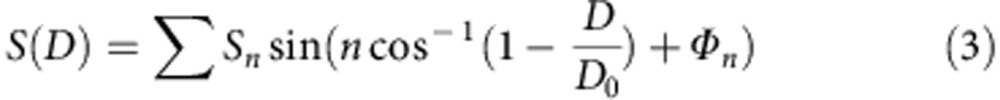


We call the relation between *D* and *S* the s-SNOM interaction curve. Figure 1b shows the s-SNOM waveform Fourier synthesized from lock-in demodulation. Figure 1c shows the reconstructed s-SNOM interaction curve that represents the total scattering signal *S* as a function of tip–sample distance *D*. However, the s-SNOM interaction curve reconstructed from all harmonics contains not only the contribution from near-field scattering but also from the undesirable background. As Fig. 1c indicates, a nonlinear increase in the signal is observed when the tip is close to the sample within 50 nm, while the overall full-range behaviour of the s-SNOM interaction curve is dominated by a linear increase of the scattering signal when the tip approaches the sample, which is a characteristic of non-near-field background. For this particular s-SNOM interaction curve, a linear fit of the background in the region of large tip–sample distance (dashed line) can be used to remove it. However, the strength and phase of the non-near-field background are not fixed values, due to variations in optical alignment and tip–sample geometry from experiment to experiment. The total waveform sometimes has a complex shape that invalidates an approach that uses a simple linear fit. Supplementary Fig. 5 shows another experimentally collected, Fourier-synthesized waveform. This waveform does not exhibit a clean oscillatory profile, and the background cannot be accurately fitted. Further analysis is needed to process the s-SNOM interaction curves to recover a pure near-field contribution.

**Figure 2:**
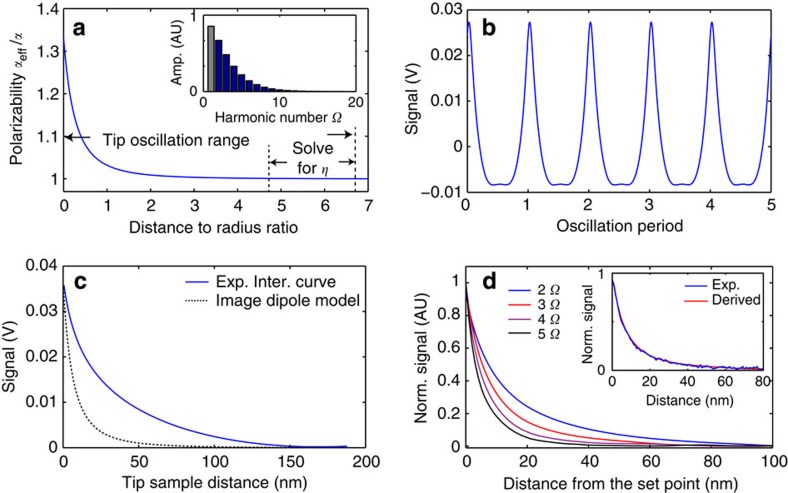
Reconstructed interaction curve and derived approach curves. (**a**) Numerical simulation of the relation between the amplitude of effective polarizability *α*_eff_ and the tip–sample distance represented as the dimensionless quantity—ratio of distance to tip radius. The simulation is carried out with the point dipole model, assuming that the tip is made of platinum and substrate is gold. The simulation of the lock-in response based on the calculated polarizability is shown in the inset. (**b**) Near-field waveform reconstructed from same data as Fig. 1b,c. The contribution of the first harmonic is found to be 0.262. (**c**) Interaction curves reconstructed from (**b**). The point dipole model is plotted with the dashed curve for reference. The tip radius used in the simulation is 28 nm as found by the AFM tip quantification procedure. (**d**) Normalized approach curves derived from the second to the fifth harmonic from the interaction curve. The probe peak-to-peak oscillation amplitude is chosen to be 80 nm. A comparison between the derived third harmonic approach curve and the experimentally acquired third harmonic approach curve with the same probe peak-to-peak oscillation amplitude is shown as the inset in (**d**).

Various sources contribute to the background in s-SNOM: direct reflection from the tip and cantilever, diffusive reflection from tip holder, scattering by the rough surface of the sample and so on. These sources can be categorized into two types: DC signals that are independent of tip oscillation frequency *Ω*, and AC signals that are dependent on the frequency *Ω*. The DC far-field signals are independent of the reference frequency, and therefore are excluded by the lock-in demodulation. For the AC far-field signals, because of the small tip oscillation amplitude of ∼100 nm, compared with the large size of the focus of the infrared light (>20 μm) at the tip apex, the probe tip in harmonic mechanical oscillation scatters a nanoscopically uniform region of the incident infrared field. Thus, the AC signal of the far-field contribution is harmonic and present only in the first harmonic demodulation. The progressive component of the near field has a range of up to the wavelength *λ*. In the mid-infrared frequency, where the wavelength is several microns, the progressive component of the sample near field within ∼100 nm from the sample surface can be treated with a linear position dependence. Therefore, it is present only in the first harmonic demodulation. On the other hand, the near-field interaction of the tip and sample generates the scattering signal at various harmonics of the tip oscillation frequency. Figure 2a shows the numeric simulation of scattering amplitude vs tip–sample distance with point dipole model of the near-field interaction^8^,^9^. The rapid nonlinear increase at a close proximity between tip and sample leads to anharmonicity in corresponding near-field scattering amplitude. While the near-field contribution dominates non-fundamental, higher harmonics signals (*n*≥2), it also contributes to the first harmonic signal (the grey bar of inset of Fig. 2a). Therefore, the question for the proper model is how to quantify the contribution from the near-field interaction to the first harmonic s-SNOM signal.

To answer this question, we define a contribution coefficient *η* to represent the fraction of the first harmonic component that arises from the near-field interaction. The near-field waveform *S*_NF_(*t*) from the Fourier synthesis can then be expressed as


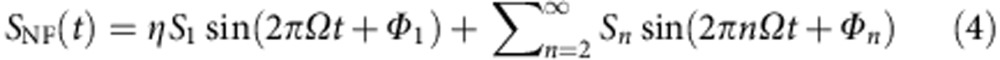


With the harmonic motion of the tip that satisfies equation (2), the interaction curve *S*_NF_(*D*) is adjusted accordingly:


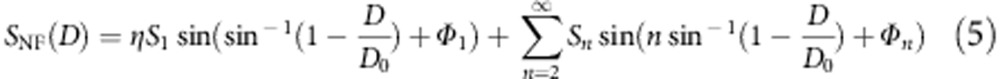


To determine *η*, we use a phenomenological behaviour of the near-field interaction, that is, the near-field scattering signal approaches zero when the tip and sample are brought to large separations. As the results from the numeric simulation in Fig. 2a show, when the tip is positioned away from the surface by several tip radii, the near-field interaction is suppressed. Under this condition, the near-field signal should remain negligible. We propose a criterion based on the tip radius to quantify a threshold in the tip–sample separation where the near-field signal becomes negligible. The tip radius is a relevant metric, because the length scale of the field decay at the tip apex is determined by the field localization, which has a direct dependence on the tip radius^1^. According to the numeric simulation, the threshold should be set at *D*/*r*>4. For a metal-coated probe tip with ∼30 nm radius, a tapping mode AFM operation with a peak-to-peak oscillation amplitude of 150 nm satisfies such a requirement at the upper turning point of the tip oscillation. This criterion creates a mathematical requirement that the absolute magnitude of the slope of *S*_NF_(*D*) should remain small in a region where the tip is away from the sample by more than four times of the tip radius *r* up to the upper turning point, where *D*=2*D*_0_, assuming *D*_0_>2*r*. Thus, this condition means the slope of the *S*_NF_(*D*) is minimized, as the near-field signal becomes insensitive to the change of tip–sample distance *D* when *D* is large. This phenomenological behaviour creates a constraint for equation (5) that can be used to solve for *η*. We define a metric *M* that is the sum of the square of the derivatives of interaction curve 
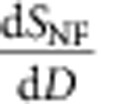
, in a region from *D*=4*r* to *D*=2*D*_0_ (the upper turning point).


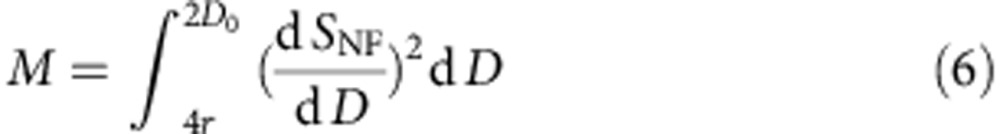


Minimization of metric *M* respect to *η* provides a suitable choice for *η* that satisfies the aforementioned constraint. The value of *η* can be obtained either analytically or numerically as the solution of 
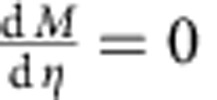
 with 
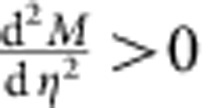
. Note that the lower bound of *D* for the integration region of *M* can be any relevant value greater than 4*r*, so long as the near-field interaction becomes negligible beyond this distance. With the coefficient *η* found from the minimization of metric *M* in equation (6), the reconstructed pure near-field waveform *S*_NF_(*t*) can be calculated. Figure 2b shows the reconstructed near-field waveform from the same experimental data as Fig. 1b, with *η*=0.262. The near-field waveform exhibits periodical and anharmonic behaviours. The near-field interaction curve is calculated with equation (5) and shown as the blue curve in Fig. 2c. To distinguish the raw s-SNOM interaction curve that contains non-near-field background, we call the reconstructed curve from equation (5) the near-field interaction curve, or simply, the interaction curve. The interaction curve exhibits a nonlinear decay as the tip–sample distance *D* increases. Compared with the near-field interaction described by the point dipole model (dashed line), the reconstructed interaction curve shows a somewhat slower decay. Such a deviation is likely because of the probe tip acting as a finite dipole in the near-field scattering process rather than as a point dipole^10^.

The interaction curve represents the actual distance dependence of the near-field scattering signal from the tip–sample interaction. The traditional approach curves can be calculated from the interaction curves (see Supplementary Fig. 6). Figure 2d shows the derived approach curves from the second to the fifth harmonics from the experimentally obtained interaction curve, assuming a tip oscillation peak-to-peak amplitude of 80 nm. A trend of decreasing decay length from the second to the fifth harmonics is observed, which agrees with the trend shown in experimentally collected approach curves (Supplementary Fig. 1). The inset of Fig. 2d shows a comparison of the derived third harmonic approach curve with a directly measured third harmonic approach curve. The derived approach curve clearly coincides with the experimental approach curves, demonstrating the accuracy of the reconstruction of the near-field interaction. Note that the derived approach curves exhibit faster decay than the interaction curve, despite both being derived from the same data. This is because the approach curves of high harmonic demodulation are based on nonlinearity of the near-field interaction within the range of tip oscillation rather than the magnitude of the near-field interaction.

### Vertical response of surface phonon polaritons of BNNTs

Described reconstruction procedure enables a direct study of vertical interactions of metallic tip and sample. In this article, we first explore an application of our method to analyse the vertical response of surface phonon polaritons of BNNTs. Surface phonon polariton is a type of a surface wave that contains a bound electromagnetic field with lattice phonon vibrations^33^. The presence of surface polaritons contributes to the optical properties of the materials, leading to an increase in local electric field amplitude and an increase in optical density of states. Boron nitride and boron nitride nanotubes have been found to support surface phonon polaritons^18^,^19^. One currently underexplored aspect is the experimental characterization of vertical field distributions of SPhP in polariton-supporting materials, which we will investigate here.

**Figure 3:**
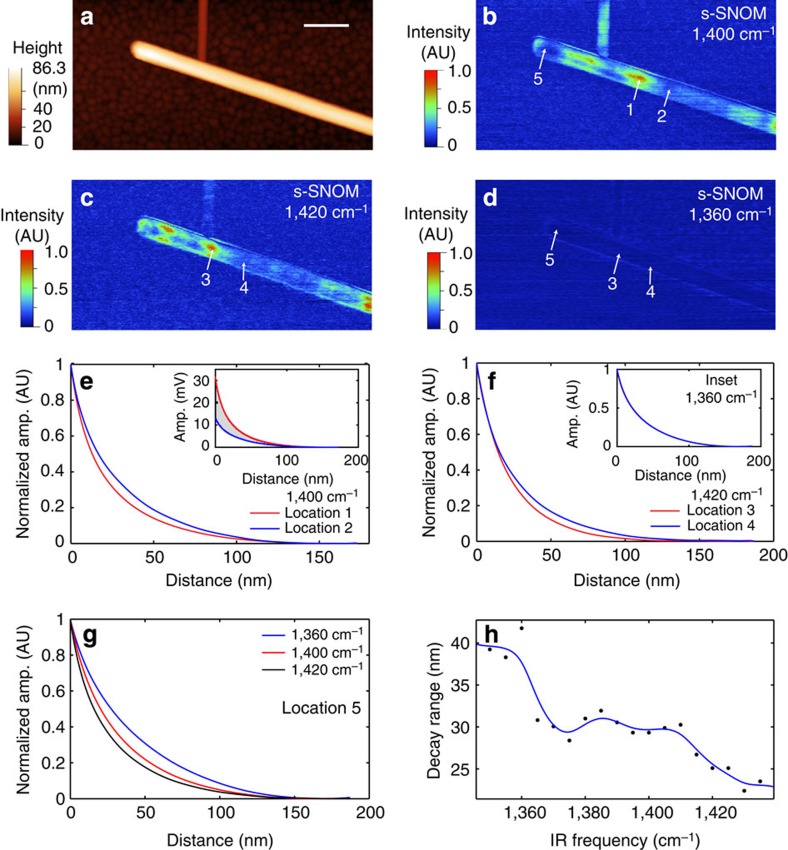
s-SNOM images and interaction curves of BNNTs. (**a**) Topography of BNNTs from AFM. Scale bar, 300 nm. The diameter of the nanotube is ∼70 nm. (**b**) s-SNOM image at 1,400 cm^−1^. (**c**) s-SNOM image at 1,420 cm^−1^. (**d**) s-SNOM image at 1,360 cm^−1^. (**e**) Interaction curves taken at locations 1 (red) and 2 (blue) at 1,400 cm^−1^. The locations are marked in **b**. The minimum and maximum amplitudes are normalized to 0 and 1, respectively. The interaction curves at locations 1 and 2 without normalization are shown in the inset of **e**. (**f**) Interaction curves at locations 3 (red) and 4 (blue) at 1,420 cm^−1^. The interaction curves at 1,360 cm^−1^ for locations 3 and 4 are shown in the inset. They have identical shapes and overlap after normalization. (**g**) The interaction curves at location 5 at infrared frequencies of 1,360, 1,400 and 1,420 cm^−1^. (**h**) The values (black dot) of 1/*e* decay length for the interaction curves at location 5 with infrared frequencies from 1,350 to 1,435 cm^−1^. Blue curve is shown as a guide.

Boron nitride nanotubes were spectroscopically imaged with s-SNOM under *π*/2 phase homodyne condition^34^ with infrared frequencies tuned within the window of surface phonon polariton active frequencies. s-SNOM with *π*/2 phase homodyne condition maps the imaginary parts of the s-SNOM signal of the sample. The s-SNOM image of total amplitude is shown in Supplementary Fig. 7 as a reference. The topography of the BNNTs placed on a rough gold substrate is shown in Fig. 3a. The diameter of the nanotube is ∼70 nm. The s-SNOM image of the same area at infrared frequency of 1,400 cm^−1^ is shown in Fig. 3b. The bright and dark nodes are the manifestation of SPhPs propagating along the tube. The s-SNOM image at infrared frequency of 1,420 cm^−1^ (Fig. 3c) shows shifted nodal patterns, which are the manifestation of the dispersion of SPhPs under high infrared frequency^19^. Below the SPhP active frequency, s-SNOM of 1,360 cm^−1^ is shown in Fig. 3d as a reference, which does not show appreciable s-SNOM signal, because of the absence of SPhPs. Five locations on the BNNT are chosen to acquire the near-field interaction curves. Locations 1 and 2 are in the bright and dark regions of SPhPs on the 1,400 cm^−1^ s-SNOM image. Locations 3 and 4 are in the bright and dark regions of the 1,420 cm^−1^ s-SNOM image. Location 5 is at the terminal of the BNNT. The interaction curves of locations 1 and 2 at 1,400 cm^−1^ are shown in Fig. 3e. The interaction curves are normalized by the maximal amplitude to show the difference in the decay length at these two locations. Location 1 of the bright spot shows faster vertical decay from the interaction curve than location 2 of the dark spot. The interaction curves without normalization are shown in the inset. Interaction curves at locations 3 and 4 with 1,420 cm^−1^ infrared frequency are shown in the Fig. 3f. Similarly, a difference in the decay length was also observed, though with different shapes. In contrast, the interaction curves at locations 3 and 4 of 1,360 cm^−1^ infrared frequency show nearly identical shapes after normalization on the maximal amplitude. The presence of SPhPs at 1,400 and 1,420 cm^−1^ leads to the difference on vertical decay length between the bright and dark regions of the s-SNOM image of the BNNT. Interaction curves for excitation at 1,360, 1,400 and 1,420 cm^−1^ at the terminal of the BNNT (location 5) are shown in Fig. 3g. The interaction curve at 1,360 cm^−1^ shows the longest decay length, the 1,400 cm^−1^ intermediate and the 1,420 cm^−1^ the shortest decay length. Figure 3h shows the 1/*e* amplitude decay length (distance for the amplitude to fall to 1/*e*) of the interaction curves taken at different infrared frequencies from location 5. The decay length shows an overall trend to decrease as the infrared frequency increases. In the non-SPhP region of BN, that is, below the phonon polariton frequency, the vertical decay length of the interaction curves for BN is longer than decay lengths in the SPhP active region (that is, starting from 1,365 cm^−1^). In the SPhP active region, the decay length shows an overall decreasing trend from around 30 nm to below 25 nm.

**Figure 4:**
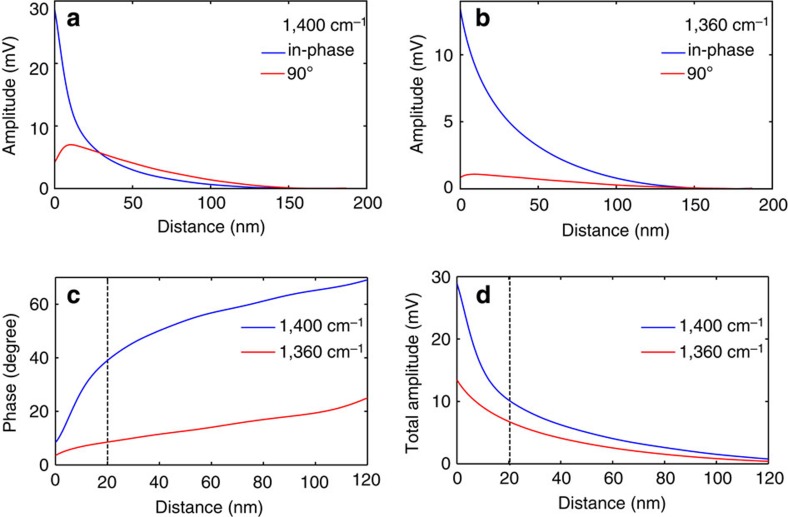
Interaction curves of the BNNT terminal under quadrature homodyne conditions. (**a**) Boron nitride interaction curves with in-phase homodyne (blue) and 90° phase homodyne condition (red) at the terminal of the BN nanotube as marked by location 5 in Fig. 3b. The infrared frequency is chosen at 1,400 cm^−1^ (**b**) Interaction curves with in-phase homodyne (blue) and 90° phase homodyne condition (red) at the same location as **a** at 1,360 cm^−1^. (**c**) The distance dependence of the phase of the near-field interaction at 1,400 cm^−1^ (blue) and at 1,360 cm^−1^ (red). The phase is calculated from quadrature vectors displayed in (**a**,**b**). (**d**) The distance dependence of the vector amplitude of the near-field interaction at 1,400 cm^−1^ (blue) and 1,360 cm^−1^ (red). The dashed line is performed at 20 nm in both (**c**,**d**).

The phase controlled homodyne technique allows acquisition of the interaction curves under different phase conditions^35^. Figure 4a shows the 1,400 cm^−1^ interaction curves taken at location 5 of the BNNT with in-phase (blue curve) and 90° out of phase (red curve) homodyne condition. It can be seen that the in-phase interaction curve shows a quick decrease with increasing tip–sample distance over 180 nm range of interest. On the other hand, the 90° homodyne interaction curve shows a slow increase of signal with a reduced tip–sample distance, followed by a drop in signal when the tip–sample separation <10 nm. The maximum amplitude of the interaction curve of 90° homodyne condition is 24.5% of that of the in-phase homodyne condition. As a comparison, the interaction curves at 1,360 cm^−1^ infrared frequency with in-phase and 90° homodyne conditions are shown in Fig. 4b. The maximum amplitude of the interaction curve of 90° homodyne condition at 1,360 cm^−1^ is 8.0% of that of the in-phase homodyne condition. Just as in the case of 1,400 cm^−1^, a decrease in signal is observed at 1,360 cm^−1^ at a close range of ∼9 nm. The acquisition of quadrature interaction curves allows the calculation of the phase of the near-field interaction. Figure 4c shows the relations between the phase of near-field interaction and tip–sample distance at 1,400 cm^−1^ (blue) and 1,360 cm^−1^ (red). A drastic change in dependence of the near-field interaction phase angle occurs at a tip–sample distance of ∼20 nm in the case of 1,400 cm^−1^. The phase changes 30.6° from the tip–sample in contact to a distance of 20 nm. In comparison, at 1,360 cm^−1^ infrared frequency, the interaction phase shows a slow varying trend, increasing by 5.0° from contact to the distance of 20 nm. The drastic change of phase angle behaviour at 1,400 cm^−1^ is due to the contribution from the surface phonon polaritons. Figure 4d shows the vector sum of interaction curves with the in-phase and 90° phase homodyne conditions. Again, at the SPhP active frequency of 1,400 cm^−1^, a drastic increase of the total interaction amplitude is observed below 20 nm of tip–sample distance. In comparison, at 1,360 cm^−1^, the trend shows a moderate increase, much smaller than at 1,400 cm^−1^. The behaviour of the total near-field amplitude indicates the appearance of an additional contribution to the near-field scattering at 1,400 cm^−1^ compared to 1,360 cm^−1^.

The presence of phonon polaritons leads to a significant tip–sample interactions that is associated with amplitude and phase change. Taubner *et al*.^29^ have explored phase and amplitude behaviours of SPhPs of SiC with the acquisition of approach curves with two quadrature homodyne conditions. They have visualized with a polar plot the s-SNOM amplitude and phase relations that are a manifestation of tip phonon polariton interaction. Here, we can also convert the interaction curves to approach curves with the same procedure. The polar plots of derived s-SNOM amplitude and phase (Supplementary Fig. 8) show s-SNOM amplitude and phase relations for BN that are very similar curves to SiC reported by Taubner *et al*.^29^, suggesting similar tip-SPhP interaction mechanism. It is worth mentioning that the phase angles derived from the approach curves are considerably larger than the phase angles obtained directly from the interaction curves, despite both being derived from the same measurement data. The observed difference is due to different signal generation mechanisms.

## Discussion

Measurements of the interaction curve suggest that the near-field signals from the polaritonic materials come from two sources. The first one is the intrinsic near-field scattering based on the modification of the tip polarizability from the redistribution of charges, carriers or dipoles in the sample. At 1,360 cm^−1^, where the SPhP is absent, this contribution is dominant. The decay length at 1/*e* amplitude is found to be ∼40 nm for BN between 1,350 and 1,360 cm^−1^. The second contribution is from the local bound electromagnetic field component of the polaritons. The sharp metallic tip converts the field component of SPhPs to detectable scattering light field. The bound field of SPhPs is close to the polariton-supporting surface of BN, thus leading to an additional increase of near-field scattering when tip is in close proximity. Because of the presence of the short-range surface bound component, the measured decay length for BN in the SPhP active frequencies shortens compared to the situation where the polariton is absent. The trend of the decreasing decay length vs the frequencies (Fig. 3f) suggests that the field components of SPhP become stronger bound to the BN surface at a higher infrared frequency. If we assume that the bound-field component accounts for the difference between the bright and dark regions of the BNNT (the difference is marked with grey area in the inset of Fig. 2e), we found a vertical 1/*e* decay length of 17 nm for the bound-field component of SPhPs. This range is consistent with the range of the significant change of phase angle (Fig. 4c), and the drastic increase in the total signal amplitude (Fig. 4d).

The vertical 1/*e* near-field decay length is also dependent on the geometry of the nanotube. Supplementary Fig. 9a shows the frequency dependence of the vertical 1/*e* near-field decay lengths at a terminal of a BNNT having 55 nm diameter. The frequency vs decay length shows a somewhat different shape from that of Fig. 3h. The minimum 1/*e* vertical near-field decay length from measurement shown in Supplementary Fig. 9a is about 14 nm, in contrast to 22 nm as shown in Fig. 3h. This greater confinement is likely due to the reduced diameter of the nanotube.

The proposed reconstruction does not assume any particular form for the tip–sample interaction model. The reconstruction of interaction curves relies on two types of readily measureable quantities: the time-varying scattering signal from the detector, and the parameters of mechanical oscillation of the tip. The only assumption in the reconstruction is that the near-field signal amplitude varies slowly when the tip is close to the upper turning point of its mechanical oscillation. This assumption is satisfied with the operation of tapping mode at large mechanical oscillation amplitudes. In our measurement, a peak-to-peak oscillation amplitude of ∼187 nm was used. Given the fact that the radius of the tip is found to be 28 nm through a standard tip shape characterization procedure, such a large mechanical oscillation in tapping mode means the changes in amplitude of the near-field signal are negligible when it is separated by the distance exceeding tip radius by a factor of 4.

The reconstruction of the near-field interaction curves has several advantages over the approach curves for studying the vertical characteristic of near-field interaction. Firstly, the interaction curve takes into consideration all harmonic responses in a single curve. Its shape is not affected by the tapping mode amplitude set point (see Supplementary Fig. 10), since it represents the core response of the tip–sample near-field interaction. In comparison, the shape of the approach curves depends on the choice of harmonic demodulations and the choice of tip tapping amplitude (see Supplementary Fig. 1). Quantitative analysis based on the approach curve would have to consider the lock-in order and tip oscillation amplitude. Secondly, measuring interaction curves does not require explicit tip–sample distance ramp performed in the tapping mode. This feature leads to a reduction in time to capture data on the near-field interaction. Several tens of milliseconds of lock-in time constants are enough to collect harmonics with sufficiently low noise for the reconstruction of interaction curves. In contrast, to acquire approach curves, tip needs to slowly approach towards or retract from the sample taking time on the order of seconds or longer, as at each tip–sample separation, a sufficient amount of time is required to obtain lock-in demodulations. Thirdly, the interaction curve contains less low-frequency noise than the approach curve. During acquisition of approach curves, the feedback of the AFM is switched off. As a consequence, the tip–sample separation is vulnerable to fluctuations or drifts, which correspond to low-frequency noise. In contrast, in the collection of interaction curve, the feedback loop is closed and the fluctuations or drift of the average tip–sample distance are actively compensated. Lastly, the interaction curve can be used to derive the approach curves, thus providing comparable information (Supplementary Fig. 11 shows an additional example of derivation of approach curve from an interaction curve on the BNNT sample). In this regard, the method of interaction curves can supersede the method of approach curves in applications^30^,^36^ that use acquisition of approach curves.

One potential limitation of the reconstruction method comes from possible anharmonic mechanical oscillations of the probe in the tapping mode. The distance between the tip and sample is derived based on the assumption that the tip oscillates harmonically. Anharmonic mechanical oscillation would lead to a systematic error in the distance between tip and sample and artefacts in non-fundamental harmonic lock-in signals. In this regard, real-time tip oscillation signals should be monitored to ensure its harmonic motion (Supplementary Fig. 12).

In summary, we have developed an experimental method for reconstruction of vertical near-field interaction in s-SNOM. The interaction curves are obtained experimentally to investigate the amplitude and phase of the tip–sample interaction. As the first application of the method, we investigated the vertical near-field responses of surface phonon polaritons in boron nitride nanotubes. The near-field response of boron nitride in the surface phonon polariton active region is found to contain a short-range field component confined to <20 nm within the surface and is associated with a considerable change of the near-field phase. The reconstruction method is expected to be generally applicable to characterize the near-field response of any sample, in particular, polaritonic materials, such as graphene and hexagonal boron nitride^37^. This method shall provide information on energy transfer mechanism through the bound electromagnetic near field of nanostructures^38^. The access to vertical field component will benefit the nanoscale characterization of metamaterials^39^,^40^. Further development of this method will be on the integration of the reconstruction method with imaging to achieve tomography. This approach will provide spatially resolved images of vertical field behaviour for nano-optical materials and structures, thus providing extensions to the current field mapping capabilities of s-SNOM^41^.

## Methods

### Experimental setup

The experiment was carried out on a custom-built s-SNOM setup. An AFM with stage scanning capability (Bruker Multimode) was operated in the tapping mode with a platinum-coated scanning probe tip (DPE-14 Mikromasch). A quantum cascade laser (MIRcat, Daylight Photonics) was used as the light source to provide frequency-tunable mid-infrared radiation. A 50:50 infrared beam splitter in a Michelson interferometer was used to split the infrared radiation into two beams. The first beam was focused to the tip region of the AFM with a parabolic mirror (effective focal length of 25 mm). The probe tip locally enhanced the infrared field at its apex, and the presence of a sample underneath the tip apex modified the light scattered by the probe. In the s-SNOM measurement, the scanning probe tip was vertically oscillating in the tapping mode with a frequency of ∼151 kHz, as a result, the scattered light was modulated at the harmonics of the oscillating frequency. The same parabolic mirror was used to collect and collimate the scattered light. The second beam was guided and reflected by an end mirror with a position feedback, to adjust and maintain the optical phase difference of the Michelson interferometer according to the method described in literature^35^. The scattered light from the tip region was recombined with the reflected beam to achieve interferometric homodyne amplification. The combined infrared field was focused onto a mercury cadmium telluride detector (KolmarTech KLD) to be converted into voltage signals.

### Multi-harmonic lock-in acquisition and homodyne detection

A multi-channel lock-in amplifier (HF2Li Zurich Instruments) was used to demodulate the signal and acquire signal at multiple harmonics with a program written in Labview (National Instrument). Both amplitude and phase of lock-in demodulations were recorded for the reconstruction of interaction curves with a program written in MATLAB (Mathworks). The s-SNOM images were taken with ***π***/2 phase homodyne technique described in literature^34^, which maps the imaginary part of the near-field signal. The ***π***/2 phase homodyne technique uses the non-resonant gold substrate as a phase reference to set the homodyne phase to ***π***/2, by minimizing lock-in non-fundamental demodulation signal from the non-resonant gold substrate through adjustment of the position of the reference mirror. The homodyne phase for the collection of lock-in harmonics for interaction curve reconstruction was chosen to maximize the fourth harmonic demodulations. The 90° homodyne condition in Fig. 4 was set by translating the reference mirror for 1/8 of the wavelength of the infrared light from the maximized location for the fourth harmonic s-SNOM signal.

## Additional information

**How to cite this article:** Wang, L. & Xu, X. G. Scattering-type scanning near-field optical microscopy with reconstruction of vertical interaction. *Nat. Commun.* 6:8973 doi: 10.1038/ncomms9973 (2015).

## Supplementary Material

Supplementary Information Supplementary Figures 1-12 and Supplementary References.

## References

[b1] Novotny L. & Stranick S. J. Near-field optical microscopy and spectroscopy with pointed probes. Annu. Rev. Phys. Chem. 57, 303–331 (2006). 16599813 10.1146/annurev.physchem.56.092503.141236

[b2] Huang B., Bates M. & Zhuang X. Super resolution fluorescence microscopy. Annu. Rev. Biochem. 78, 993 (2009). 19489737 10.1146/annurev.biochem.77.061906.092014 PMC2835776

[b3] Betzig E. . Imaging intracellular fluorescent proteins at nanometer resolution. Science 313, 1642–1645 (2006). 16902090 10.1126/science.1127344

[b4] Willig K. I., Rizzoli S. O., Westphal V., Jahn R. & Hell S. W. STED microscopy reveals that synaptotagmin remains clustered after synaptic vesicle exocytosis. Nature 440, 935–939 (2006). 16612384 10.1038/nature04592

[b5] Zenhausern F., O'boyle M. & Wickramasinghe H. Apertureless near-field optical microscope. Appl. Phys. Lett. 65, 1623–1625 (1994).

[b6] Bachelot R., Gleyzes P. & Boccara A. Near-field optical microscope based on local perturbation of a diffraction spot. Opt. Lett. 20, 1924–1926 (1995). 19862204 10.1364/ol.20.001924

[b7] Knoll B. & Keilmann F. Near-field probing of vibrational absorption for chemical microscopy. Nature 399, 134–137 (1999).

[b8] Knoll B. & Keilmann F. Enhanced dielectric contrast in scattering-type scanning near-field optical microscopy. Opt. Commun. 182, 321–328 (2000).

[b9] Raschke M. B. & Lienau C. Apertureless near-field optical microscopy: Tip–sample coupling in elastic light scattering. Appl. Phys. Lett. 83, 5089–5091 (2003).

[b10] Cvitkovic A., Ocelic N. & Hillenbrand R. Analytical model for quantitative prediction of material contrasts in scattering-type near-field optical microscopy. Opt. Express 15, 8550–8565 (2007). 19547189 10.1364/oe.15.008550

[b11] Formanek F., De Wilde Y. & Aigouy L. Analysis of the measured signals in apertureless near-field optical microscopy. Ultramicroscopy 103, 133–139 (2005). 15774274 10.1016/j.ultramic.2004.11.004

[b12] Amarie S. . Nano-FTIR chemical mapping of minerals in biological materials. Beilstein J. Nanotechnol. 3, 312–323 (2012). 22563528 10.3762/bjnano.3.35 PMC3343267

[b13] Huth F. . Nano-FTIR absorption spectroscopy of molecular fingerprints at 20 nm spatial resolution. Nano Lett. 12, 3973–3978 (2012). 22703339 10.1021/nl301159v

[b14] Xu X. G., Rang M., Craig I. M. & Raschke M. B. Pushing the sample-size limit of infrared vibrational nanospectroscopy: from monolayer toward single molecule sensitivity. J. Phys. Chem. Lett. 3, 1836–1841 (2012). 26291869 10.1021/jz300463d

[b15] Qazilbash M. M. . Mott transition in VO_2_ revealed by infrared spectroscopy and nano-imaging. Science 318, 1750–1753 (2007). 18079396 10.1126/science.1150124

[b16] Esteban R. . Direct near-field optical imaging of higher order plasmonic resonances. Nano Lett. 8, 3155–3159 (2008). 18788785 10.1021/nl801396r

[b17] Hillenbrand R., Taubner T. & Keilmann F. Phonon-enhanced light–matter interaction at the nanometre scale. Nature 418, 159–162 (2002). 12110883 10.1038/nature00899

[b18] Dai S. . Tunable phonon polaritons in atomically thin van der Waals crystals of boron nitride. Science 343, 1125–1129 (2014). 24604197 10.1126/science.1246833

[b19] Xu X. G. . One-dimensional surface phonon polaritons in boron nitride nanotubes. Nat. Commun. 5, 4782 (2014). 25154586 10.1038/ncomms5782

[b20] Caldwell J. D. . Sub-diffractional volume-confined polaritons in the natural hyperbolic material hexagonal boron nitride. Nat. Commun. 5, 5221 (2014). 25323633 10.1038/ncomms6221

[b21] Fei Z. . Gate-tuning of graphene plasmons revealed by infrared nano-imaging. Nature 487, 82–85 (2012). 22722866 10.1038/nature11253

[b22] Chen J. . Optical nano-imaging of gate-tunable graphene plasmons. Nature 487, 77–81 (2012). 22722861 10.1038/nature11254

[b23] Akhremitchev B. B., Pollack S. & Walker G. C. Apertureless scanning near-field infrared microscopy of a rough polymeric surface. Langmuir 17, 2774–2781 (2001).

[b24] Raschke M. B. . Apertureless near—field vibrational imaging of block—copolymer nanostructures with ultrahigh spatial resolution. ChemPhysChem 6, 2197–2203 (2005). 16208743 10.1002/cphc.200500218

[b25] Paulite M. . Imaging secondary structure of individual amyloid fibrils of a β2-microglobulin fragment using near-field infrared spectroscopy. J. Am. Chem. Soc. 133, 7376–7383 (2011). 21524071 10.1021/ja109316p

[b26] Amenabar I. . Structural analysis and mapping of individual protein complexes by infrared nanospectroscopy. Nat. Commun. 4, 2890 (2013). 24301518 10.1038/ncomms3890 PMC3863900

[b27] Berweger S. . Nano-chemical infrared imaging of membrane proteins in lipid bilayers. J. Am. Chem. Soc. 135, 18292–18295 (2013). 24251914 10.1021/ja409815g

[b28] Hauer B., Saltzmann T., Simon U. & Taubner T. Solvothermally synthesized Sb_2_Te_3_ platelets show unexpected optical contrasts in mid-infrared near-field scanning microscopy. Nano Lett. 15, 2787–2793 (2015). 25868047 10.1021/nl503697c

[b29] Taubner T., Keilmann F. & Hillenbrand R. Nanomechanical resonance tuning and phase effects in optical near-field interaction. Nano Lett. 4, 1669–1672 (2004).

[b30] Olmon R. L. . Determination of electric-field, magnetic-field, and electric-current distributions of infrared optical antennas: a near-field optical vector network analyzer. Phys. Rev. Lett. 105, 167403 (2010). 21231012 10.1103/PhysRevLett.105.167403

[b31] Stöckle R. M., Suh Y. D., Deckert V. & Zenobi R. Nanoscale chemical analysis by tip-enhanced Raman spectroscopy. Chem. Phys. Lett. 318, 131–136 (2000).

[b32] Kawai S., Hafizovic S., Glatzel T., Baratoff A. & Meyer E. Rapid reconstruction of a strong nonlinear property by a multiple lock-in technique. Phys. Rev. B 85, 165426 (2012).

[b33] Caldwell J. D. . Low-loss, infrared and terahertz nanophotonics using surface phonon polaritons. Nanophotonics 4, 44–68 (2014).

[b34] Xu X. G., Tanur A. E. & Walker G. C. Phase controlled homodyne infrared near-field microscopy and spectroscopy reveal inhomogeneity within and among individual boron nitride nanotubes. J. Phys. Chem. A 117, 3348–3354 (2013). 23465036 10.1021/jp4008784

[b35] Xu X. G., Gilburd L. & Walker G. C. Phase stabilized homodyne of infrared scattering type scanning near-field optical microscopy. Appl. Phys. Lett. 105, 263104 (2014).

[b36] Hillenbrand R. & Keilmann F. Complex optical constants on a subwavelength scale. Phys. Rev. Lett. 85, 3029 (2000). 11005995 10.1103/PhysRevLett.85.3029

[b37] Dean C. . Boron nitride substrates for high-quality graphene electronics. Nat. Nanotechnol. 5, 722–726 (2010). 20729834 10.1038/nnano.2010.172

[b38] Xu X. G. . Mid-infrared polaritonic coupling between boron nitride nanotubes and graphene. ACS Nano 8, 11305–11312 (2014). 25365544 10.1021/nn504093g

[b39] Cui T. J., Smith D. R. & Liu R. Metamaterials Springer (2014).

[b40] Bohn B. J. . Near-field imaging of phased array metasurfaces. Nano Lett. 15, 3851–3858 (2015). 25978528 10.1021/acs.nanolett.5b00692

[b41] Rotenberg N. & Kuipers L. Mapping nanoscale light fields. Nat. Photon. 8, 919–926 (2014).

