# Supplementary material for: Scattering-type scanning near-field optical microscopy with reconstruction of vertical interaction
Source: Nat Commun. 2015 Nov 23;6:8973. doi: 10.1038/ncomms9973 (PMC4673874; doi:10.1038/ncomms9973)
Supplement: Supplementary Information — Supplementary Figures 1-12 and Supplementary References. [file ncomms9973-s1.pdf]

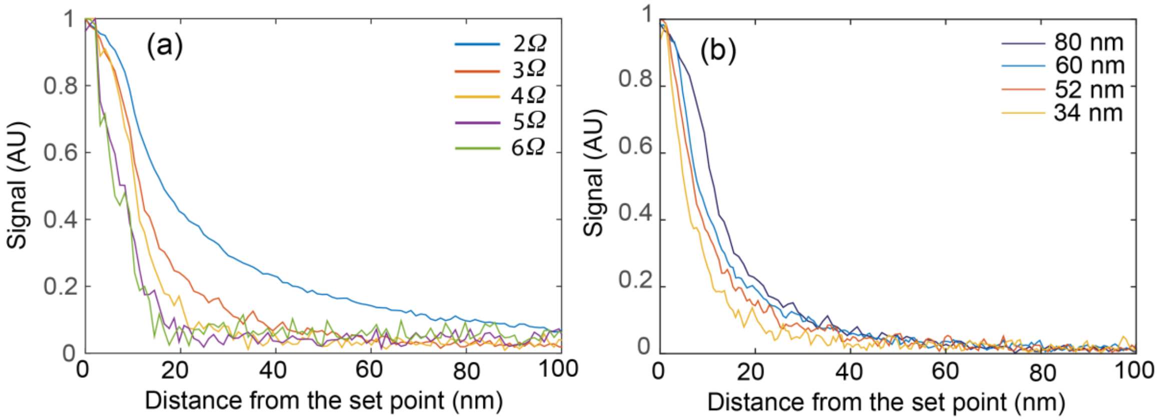

Supplementary Figure 1. Approach curves of s-SNOM

(a) Approach curves measured from the second harmonic (2Ω) to the sixth harmonic (6Ω) demodulations. The approach curves are acquired by bringing the sample toward the oscillating tip using a Z piezo stage. The lock-in demodulations at non-fundamental harmonic are extracted by the lock-in amplifier and are recorded as functions of the relative distance from the AFM height set point. The measurement was done with a 28 nm radius Pt-coated tip on a gold-coated substrate. Approach curves from different orders of lock-in demodulations exhibit different shapes. The higher harmonic approach curves exhibit shorter signal decay length than lower order harmonic approach curves. (b) Approach curves obtained from the third harmonic demodulation with the tip oscillation peak-to-peak amplitude set to 34 nm, 52 nm, 60 nm and 80 nm. The approach curves from the same order of lock-in demodulation with different tip oscillation amplitudes exhibit noticeably different shapes. This observation demonstrates that the shape of the approach curve depends on the choice of the order for lock-in demodulation and tip oscillation amplitude.

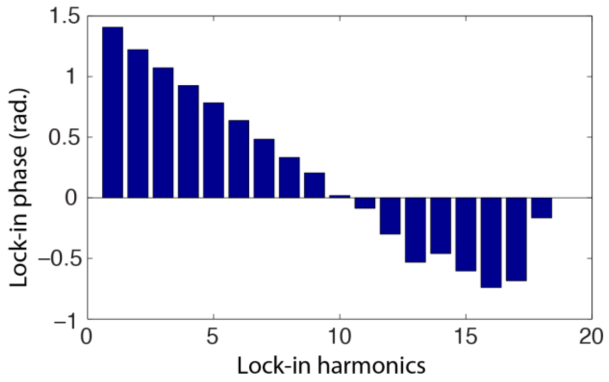

Supplementary Figure 2. Lock-in phase

Lock-in phase of 18 harmonics. They are acquired simultaneously with lock-in amplitude shown in the inset of Figure 1a of the main text. The lock-in phase is the phase difference between the Fourier components and the reference frequency. It should not be confused with the near-field phase.

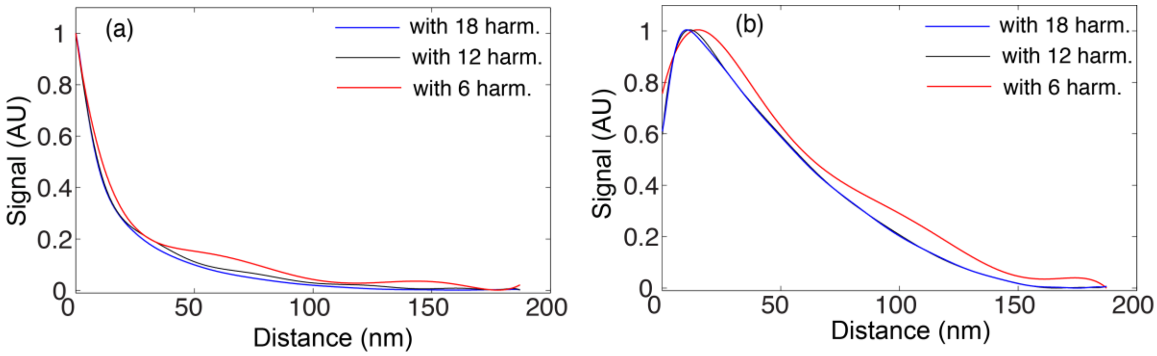

Supplementary Figure 3. Effect of the number of harmonics on the quality of the reconstructed curves

Comparisons of the results of the reconstruction of near-field interaction curves with 18 harmonics, 12 harmonics and 6 harmonics. (a) The near-field interaction curves on near-field responses with Pt-coated tip and gold substrate. The interaction curve synthesized from 18 harmonics overlaps with the interaction curve synthesized from 12 harmonics. The interaction curve synthesized from 6 harmonics shows noticeable deviations from the interaction curves of

12 or 18 harmonics, a characteristic of insufficient bandwidth used in Fourier synthesis. (b) The near-field interaction curves of the near-field responses on a terminal of BNNT with 90° homodyne phase condition at 1400 cm^{-1}, the same as Figure 4a of the main text. The interaction curve synthesized from 18 harmonics shows small but noticeable deviations from the one synthesized with 12 harmonics. The interaction curve synthesized from 6 harmonics displays clear deviations from the curve synthesized with 18 harmonics. Supplementary Fig. 2 suggests that a collection of 18 harmonics can reproduce near-field interaction curve most faithfully, whereas a collection with 6 harmonics is not sufficient for accurate reconstruction.

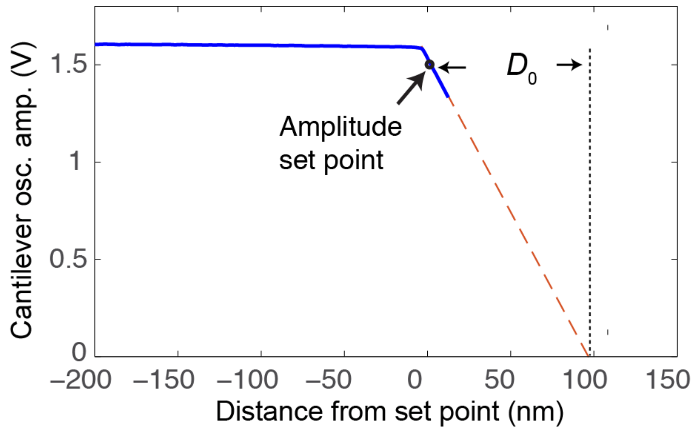

Supplementary Figure 4. Quantification of tip oscillation amplitude

Quantification of the tip oscillation amplitude in tapping mode. A ramp curve is measured and shown as the blue curve. It is done by recording the cantilever oscillation amplitude vs. the Z piezo distance from the tapping mode set point. The slope of the region where the tip amplitude exhibits linear reduction is used to extrapolate to zero amplitude (dashed line). The distance between amplitude set point to the zero amplitude is used as the half peak-to-peak oscillation amplitude, which is D_0 as defined in the main text.

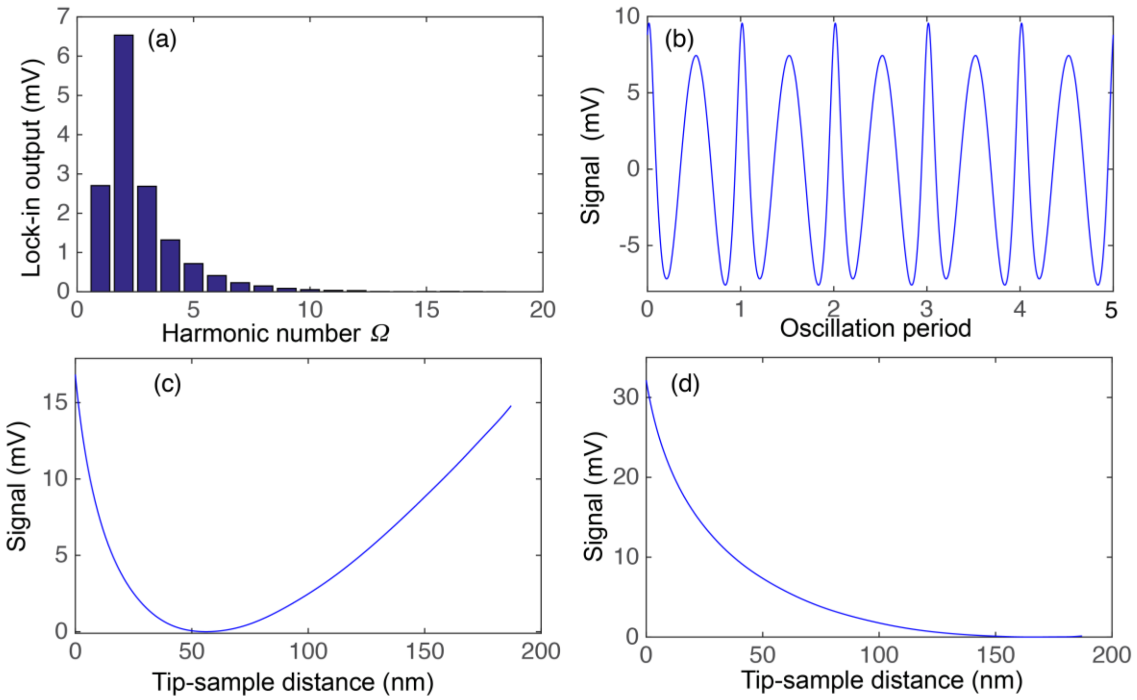

Supplementary Figure 5. An s-SNOM waveform that does not provide a good linear fit

(a) Amplitudes of lock-in harmonics. (b) Fourier-synthesized raw s-SNOM waveform does not show a simple oscillatory behavior. (c) Raw s-SNOM interaction curve does not have a clearly defined region for fitting of the linear far-field background. (d) Near-field interaction curve obtained from data in the reconstruction procedure described in the main text, it does not rely on any linear fit to remove the far-field background.

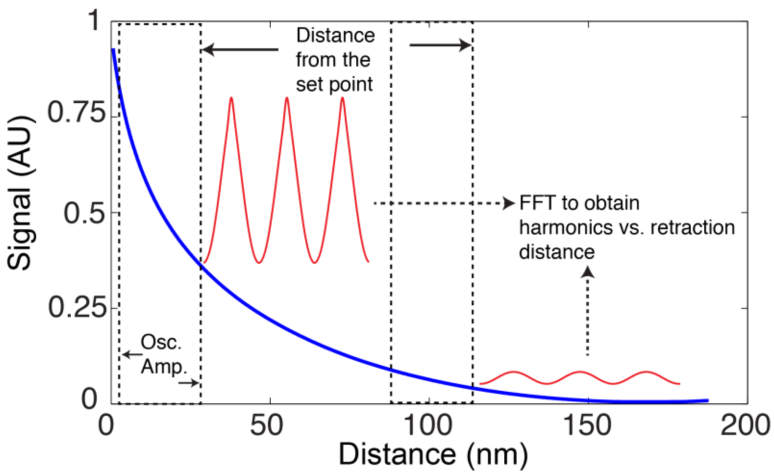

Supplementary Figure 6. Procedure to convert interaction curves into approach curves

A scheme illustrating conversion from a near-field interaction curve to an approach curve. The blue curve shows the near-field interaction curve. In construction of approach curves for multiple harmonics, the data of the near-field interaction from a window of tip-sample distances (dashed boxes) are sampled to construct the s-SNOM waveform (red curve). The size of the window is set equal to the tip oscillation amplitude. The window is shifted toward the high tip-sample distance. The shift distance is set as the tip position in the approach curves. Fourier analysis is performed on the calculated s-SNOM waveform to obtain the non-fundamental harmonics that can be plotted in the coordinates of the approach curves.

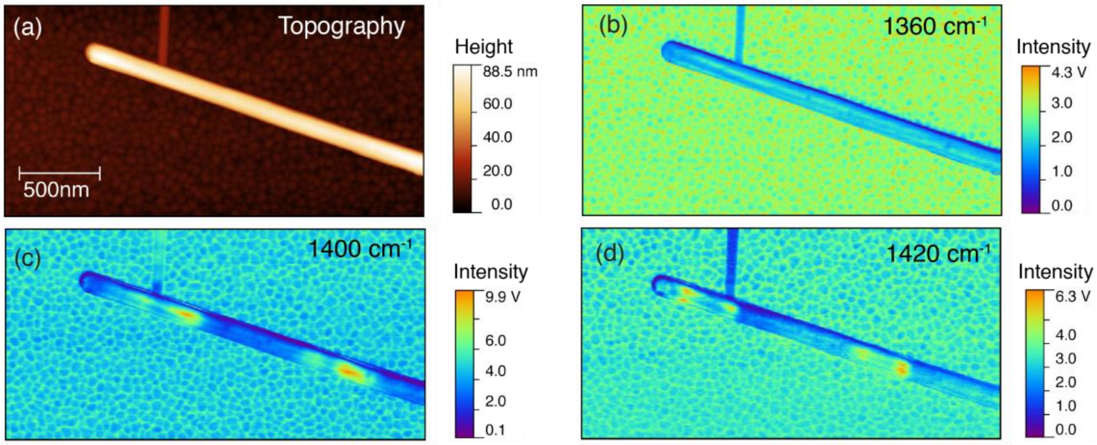

Supplementary Figure 7. Total amplitude s-SNOM image of BNNTs from dual phase homodyne

(a) AFM topography image of the BNNTs (from the same area as in Figure 3a of the main text). The s-SNOM images on amplitude of the BNNTs at 1360 cm^{-1} (b), 1400 cm^{-1} (c), and 1420 cm^{-1} (d). The images are calculated from $\pi/2$ phase homodyne condition and in-phase homodyne condition of s-SNOM with the procedures described in literature. [1,2] The 1400 cm^{-1} and 1420 cm^{-1} images show a clear nodal pattern characteristic of the surface phonon polariton. The 1360 cm^{-1} is outside the SPhP active frequency region. As a result, its corresponding image does not show amplitude variations along the BNNT.

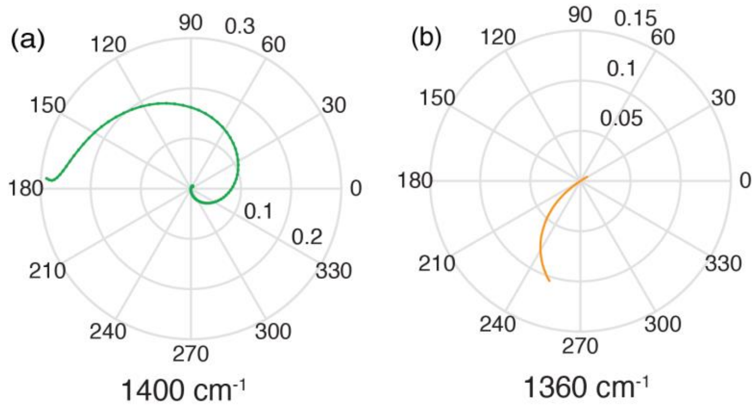

Supplementary Figure 8. Amplitude-phase polar plots from calculated approach curves

(a) s-SNOM amplitude-phase polar plot derived from near-field interaction curves of in-phase and 90° homodyne conditions at 1400 cm^{-1}, using data shown in Figure 4a of the main text. The s-SNOM phase sweeps more than 180° as the tip is approaching the sample. (b) s-SNOM amplitude-phase polar plot derived from near-field interaction curves of in-phase and 90° homodyne conditions at 1360 cm^{-1}, using data shown in Figure 4a of the main text. The s-SNOM phase changes by ~ 40°. In calculations of the polar plots, the peak-to-peak tip oscillation amplitude is set to 20 nm. The second harmonic response is used to derive the polar plots. First, corresponding approach curves at the second harmonic of in-phase and 90° homodyne conditions are calculated. Then the vector sum of the approach curves of two-quadrature components is used to calculate the amplitude and the phase used in the polar plot. The oscillation amplitude is chosen to be the same as reported by Taubner et.al.[3] The tip oscillation amplitude is also found to affect the shape of the polar plot (not shown here).

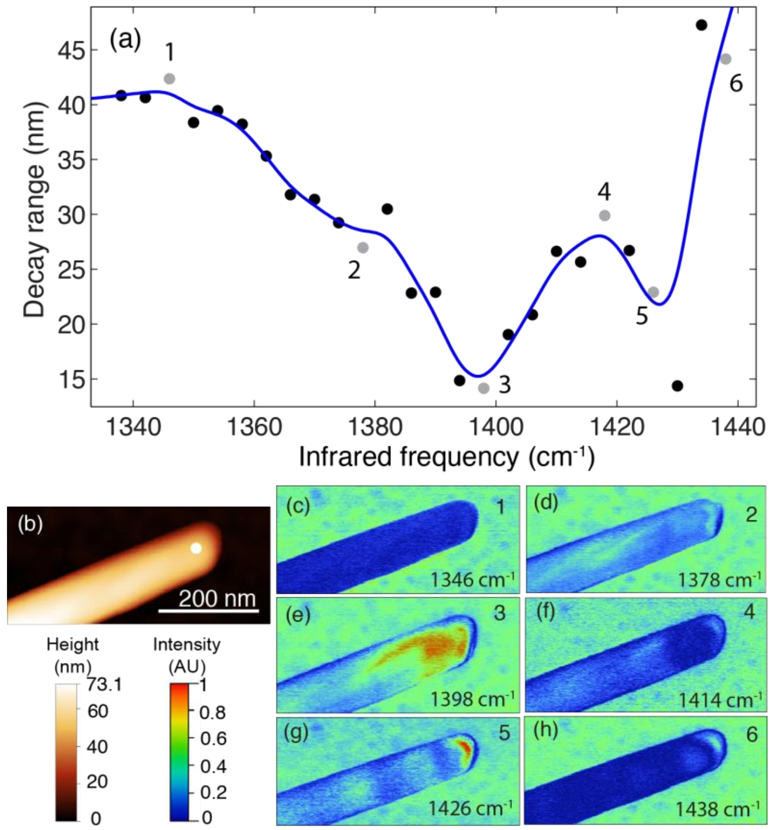

Supplementary Figure 9. Vertical near-field 1/*e* decay length of another BNNT terminal

(a) The decay length measured at the terminal of a BNNT vs. infrared frequency. Black and grey dots represent 1/*e* decay length from reconstruction of near-field interactions using experimental data for a series of infrared frequencies. (b) AFM topography image of the nanotube. The substrate is gold coated silicon wafer. The diameter of this tube is ~55 nm. The measurement location is marked by the white dot. (c-h) s-SNOM images of the same area at the infrared frequencies of 1346 cm^{-1}, 1378 cm^{-1}, 1398 cm^{-1}, 1414 cm^{-1}, 1426 cm^{-1}, and 1438 cm^{-1}. The s-SNOM images are rendered with the third harmonic of the lock-in demodulation. The s-SNOM images are calculated from $\pi/2$ phase and in-phase homodyne conditions using procedure described in literature[1,2]. The values of the decay length of these frequencies are marked with grey dots in (a). There is an inverse correlation between the s-SNOM signal at the measurement location and the vertical decay length. Low s-SNOM signals at infrared frequencies of 1346 cm^{-1} and 1438 cm^{-1} correspond to long vertical decay length in (a), while high s-SNOM signal at 1398

cm^{-1} and 1426 cm^{-1} corresponds to short vertical decay length in (a). Such an inverse correlation is due to the presence of the SPhPs. SPhPs lead to an increase of s-SNOM signal, but at the same time also strongly binds the field component to the sample surface.

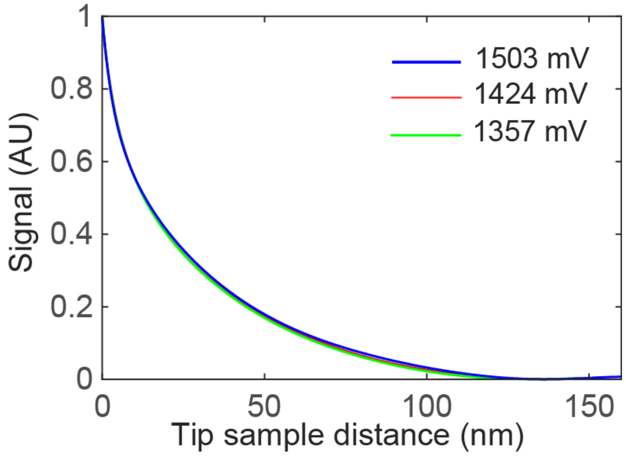

Supplementary Figure 10. Interaction curves obtained with different tapping amplitude set points.

Reconstructed interaction curve on a gold substrate obtained with three different amplitude set points. The peak-to-peak oscillation amplitudes of the tip were 162 nm, 154 nm and 146 nm for 1503 mV, 1424 mV and 1357 mV set point respectively. They overlap very well. The metric minimization region for η is chosen between 120 nm and the upper turning point of the tip. It can be seen that the choice of tapping mode set point does not affect the shape of the reconstructed interaction curve, as long as tapping amplitude is sufficiently larger than the decay length of near-field response curve.

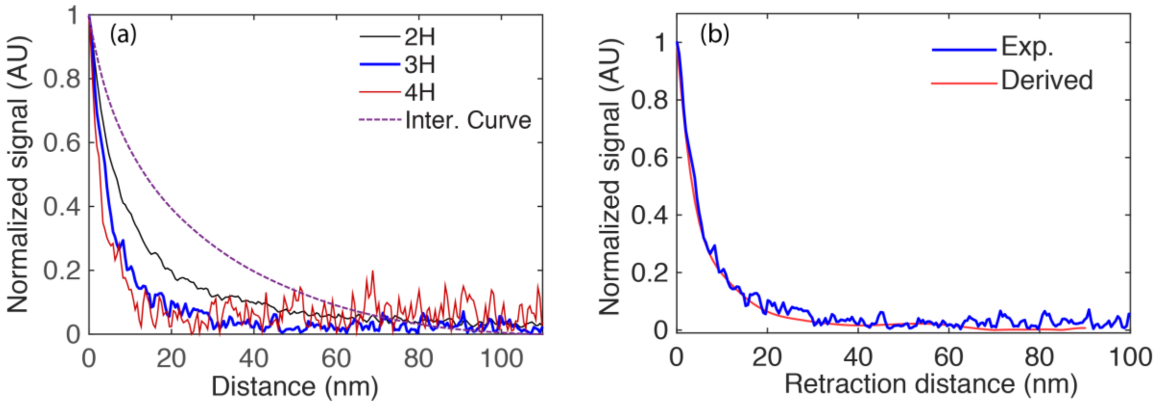

Supplementary Figure 11. Approach curves and derived approach curves of BNNT

(a) Measured approach curves from BNNT of the second (black), third (blue), and fourth (red) harmonics demodulations. The approach curve of the fifth harmonic is very noisy and not shown here. The peak-to-peak tapping amplitude in the approach curve collection is set to 48 nm. The reconstructed interaction curve is shown as the magenta dashed curve for comparison. The interaction curve shows slower decay than the approach curves. This observation on BNNT is similar to the measurement on the gold substrate shown in the main text. (b) The derived approach curve for the third harmonic demodulation (red) and the experimentally measured approach curve for the third harmonic demodulation (blue). The peak-to-peak amplitude for the simulated curve is chosen to be the same as in the experiment. The experimentally collection and the derived approach curves overlap well. The procedure is described in Supplementary Fig. 6.

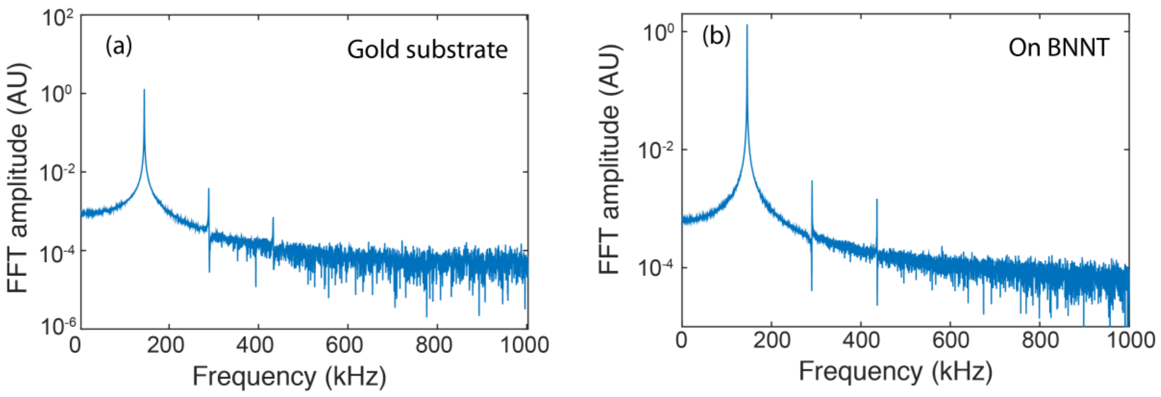

Supplementary Figure 12. Estimation on the anharmonicity of the tip mechanical oscillation (a) Fourier transform of the tip vertical deflection signal when the probe is operated in tapping mode on a gold substrate. The tip peak-to-peak oscillation amplitude is ~140 nm. The amplitude of frequencies by the Fourier transform is plotted with logarithmic scale. Amplitude of the second and third harmonic is about 0.3% and 0.06% of the amplitude of the fundamental oscillation. (b) Fast Fourier transform of the tip vertical deflection signal when the probe is operated in tapping mode on a BNNT. Amplitude of the second and third harmonic is about 0.23% and 0.011% of the amplitude of the fundamental oscillation. It can be seen that the anharmonic mechanical motions in our measurement of the gold and BNNT samples are very small. To ensure the minimum presence of the anharmonic mechanical oscillation is important, as anharmonic mechanical oscillation can create high order demodulation signals that are detectable by the lock-in detector, as the far-field scattering or the progressive field can be modulated anharmonically. The presence of anharmonic motions can be reduced by using a stiff cantilever with high reduced mass and operated at large oscillation amplitude. For sample that exhibits strong short-range forces, or large adhesion, one would expect large deviations. A solution to this problem is either to capture the tip oscillation signal through digitization of the tip vertical deflection signal, or to use the method described by Kawai S et.al.,[4] to obtain the actual time vs. tip-sample distance relationship to replace Equation (2) of the main article, with the rest of the reconstruction procedure remaining the same.

Supplementary References

1 Kim, Z. H., Liu, B. & Leone, S. R. Nanometer-scale optical imaging of epitaxially grown GaN and InN islands using apertureless near-field microscopy. *J. Phys. Chem. B* **109**, 8503-8508 (2005).

2 Xu, X. G., Gilburd, L. & Walker, G. C. Phase stabilized homodyne of infrared scattering type scanning near-field optical microscopy. *Appl. Phys. Lett.* **105**, 263104 (2014).

3 Taubner, T., Keilmann, F. & Hillenbrand, R. Nanomechanical resonance tuning and phase effects in optical near-field interaction. *Nano Lett.* **4**, 1669-1672 (2004).

4. Kawai S, Hafizovic S, Glatzel T, Baratoff A, Meyer E. Rapid reconstruction of a strong nonlinear property by a multiple lock-in technique. Phys. Rev. B 85, 165426 (2012).
